# Insulin receptor based lymphocyte trafficking in the progression of type 1 diabetes

**DOI:** 10.14440/jbm.2018.209

**Published:** 2018-01-23

**Authors:** Michael P. Morran, Ali G. Al-Dieri, Andrea L. Nestor-Kalinoski, Richard K. Jordan, Nirdesh K. Gupta, Marcia F. McInerney

**Affiliations:** 1 Department of Medicinal and Biological Chemistry, College of Pharmacy and Pharmaceutical Sciences, University of Toledo, Toledo, OH 43606, USA; 2 Department of Surgery, College of Medicine and Life Sciences, University of Toledo, Toledo, OH 43606, USA; 3 Center for Diabetes and Endocrine Research, University of Toledo, Toledo, OH 43606, USA

**Keywords:** chemotaxis, Cre-recombinase, diabetes, insulin receptor, insulitis

## Abstract

The insulin receptor (IR) is a transmembrane receptor which recognizes and binds the hormone insulin. We describe two models that were devised to explore the role of IR over-expression on T-lymphocytes and their chemotactic motility in the progression of type 1 diabetes. FVB/NJ-CD3-3×FLAG-mIR/MFM mice were generated to selectively over-express 3×FLAG tagged murine IR in T-lymphocytes *via* an engineered CD3 enhancer and promoter construct. Insertion of the 3×FLAG-mIR transgene into FVB/NJ mice, a known non-autoimmune prone strain, lead to a minor population of detectable 3×FLAG-mIR tagged T-lymphocytes in peripheral blood and the presence of a few lymphocytes in the pancreas of the Tg+/- compared to age matched Tg-/- control mice. In order to induce stronger murine IR over-expression then what was observed with the CD3 enhancer promoter construct, a second system utilizing the strong CAG viral promoter was generated. This system induces cell specific IR over-expression upon Cre-Lox recombination to afford functional 3×FLAG tagged murine IR with an internal eGFP reporter. The pPNTlox2-3×FLAG-mIR plasmid was constructed and validated in HEK-Cre-RFP cells to ensure selective Cre recombinase based 3×FLAG-mIR expression, receptor ligand affinity towards insulin, and functional initiation of signal transduction upon insulin stimulation.

## INTRODUCTION

The insulin receptor (IR) is a transmembrane receptor known for binding and being activated by insulin [1,2]. Signaling through the IR leads to the activation of multiple downstream modulators which control events such as protein translation, autophagy, apoptosis, oxidative stress, cell mobility/chemotaxis, gene transcription, and cellular differentiation and/or cell growth [3,4]. Since the IR is such a strong mediator of cellular homeostasis it has long been studied for its potential role in numerous disease states including: Alzheimer’s disease, cancer, and both type 1 (T1D) and type 2 diabetes [4-8].

It has been shown that activated human peripheral blood lymphocytes (both CD4+ and CD8+) up-regulate surface IR expression and display increased chemotaxis toward insulin [9]. Insulin is also chemotactic for macrophages, which infiltrate into the islets of non-obese diabetic (NOD) mice [10]. Previous data from our laboratory highlights the capability of IR surface expression levels as a defining component in lymphocyte directed pathogenesis of T1D [7]. In an effort to understand the role of IR over-expression on lymphocyte chemotaxis in the progression of T1D, two unique IR over-expression models systems were developed. Understanding the mechanism driving lymphocyte migration towards the pancreas and into the insulin producing islet of Langerhans is necessary to design novel therapeutic preventatives to treat T1D.

## MATERIALS AND METHODS

### Plasmid validation

Research involving recombinant DNA use was approved by the Institutional Biosafety Committee at the University of Toledo prior to being carried out. The pNeZBCD3-3×FLAG-mIR plasmid (modified from pNeZB a gift from Dr. Lee, Mayo Clinic, MN) was used to generate FVB/NJ-CD3-3×FLAG-mIR transgenic mice. Additionally, insertion of the PPT-3×FLAG-mIR gene (approximately 4.1 Kb) into the pPNTlox2 (Dr. Howard, University of Toledo, OH [11]) backbone afforded the formation of the pPNTlox2-3×FLAG-mIR plasmid. Both plasmids were sequenced at the University of Michigan’s DNA Sequencing Core Facility (Ann Arbor, MI). Sequencing data was analyzed using FinchTV software (Geospiza Inc.) and the NCBI sequence alignment tool. Sequencing primers can be found in **Table S1**.

### Generation of FVB/NJ-CD3-3×FLAG-mIR/MFM mice

In brief, FVB/NJ-CD3-3×FLAG-mIR/MFM transgenic mice were generated at the Ohio State University’s Transgenic Core Facility (Columbus, OH) using the pronuclear injection method with DNA from the pNeZBCD3-3×FLAG-mIR plasmid. Founder tail clips were polymerase chain reaction (PCR) genotyped for presence of transgene 3×FLAG and endogenous murine β-globin. Mice were characterized as either FVB/NJ-CD3-3×FLAG-mIR/MFM transgene positive (Tg+/-) or transgene negative (Tg-/-). Mice were bred and housed in specific pathogen free conditions in the Department of Laboratory Animal Resources care facility at the University of Toledo (Toledo, OH) under an approved animal protocol.

### Peripheral blood lymphocyte analysis

Approximately 100 μl of blood was collected in 200 μl of ACD buffer (Becton Dickinson) and processed to remove all red blood cells prior to lymphocyte staining. Cells were treated with TruStain FcX affinity purified anti-mouse CD16/32 Fc block (BioLegend) and stained with DYKDDDDK (FLAG) Tag specific FITC-anti-Flag M2 mAb (Sigma Cat. #F4049) and PE-anti-mouse CD3e mAb (eBioscience Cat. #eBio500A2). Cell samples were analyzed on a BD FACSCaliber cytometer (BD Biosciences).

### Tissue immunohistochemistry and imaging

Pancreatic tissue was fixed in 10% Neutral Buffered Formalin, paraffin embedded and cut in 5 μm thick sections. Sections were H&E stained and imaged at 20 × with a Nikon Eclipse Ti microscope.

### Cell culture and transfection

HEK293T (ATCC) cells were grown in DMEM (ATCC) with 10% FBS (Atlanta Biologicals) and 1% penicillin/streptomycin (Mediatech Inc). HEK-Cre-RFP (GenTarget, Inc.) cells were grown in the same media, with puromycin (ThermoFisher) selection (0.5 μg/ml), to elicit a selective pressure to ensure Cre recombinase expression. HEK-293T (HEK-Cre-) and HEK-Cre-RFP (HEK-Cre+) cells were transfected *via* Lipofectamine 3000 (Invitrogen) and were analyzed 48 h post transfection.

### Quantitative real-time PCR analysis

Cell preparations were lysed and total mRNA was purified using the RNeasy mini kit (Qiagen). An additional DNase treatment step was carried out to remove any contaminating plasmid or nucleotide product. Total cDNA was synthesized using M-MLV Reverse Transcriptase (ThermoFisher) and quantitated *via* the BioSpec-nano (Shimadzu Biotech). Each quantitative real-time PCR (qRT-PCR) was performed in triplicate using equivalent amounts of cDNA template. Amplification was performed using Absolute qRT-PCR SYBR mastermix and a CFX96 system Thermocycler (Bio-Rad). Human Glyceraldehyde-3 phosphate dehydrogenase (hGAPDH) was used to normalize the amount of relative mRNA using the delta Ct method for quantification.

DNA qRT-PCR was carried out following transfection to validate plasmid recombination in the presence of Cre recombinase. DNA was purified using the DNeasy Blood & Tissue kit (Qiagen). DNA qRT-PCR analysis was carried out in triplicate with primers to highlight Cre recombination and ablation of the decoy Lac-Z gene. All primers used in qRT-PCR analysis are listed in **Table S2**. A schematic representation of Cre recombinase inducible gene expression of 3×FLAG-mIR in the absence (-) or presence (+) of the enzyme Cre recombinase and associated qRT-PCR primers used during recombination analysis can be found in **Figure S1**.

### Protein extraction and western blot

Total protein extractions for use in the identification of the Lac-Z gene product beta-Galactosidase (LacZ) and FLAG-tagged mIR protein were carried out in RIPA lysis buffer (ThermoFisher). Protein extractions for use in the identification of phosphorylated-insulin receptor β-subunit (phos-IRβ) protein as well as FLAG resin immunoprecipitation were carried out in Native lysis buffer (Abcam). HALT Protease inhibitor cocktail (ThermoFisher) was added to both lysis buffers. Protein concentrations were quantified by Pierce BCA Protein Assay Kit (ThermoFisher).

Protein samples were prepared in either Pierce Lane Marker Reducing or Non-Reducing Sample Buffer (ThermoFisher) prior to SDS PAGE electrophoresis. Proteins were transferred onto polyvinylidene fluoride membrane (EMD Millipore). Membranes utilized for the detection of LacZ and FLAG were blocked with 5% non-fat dry milk, while membranes utilized for phos-IRβ detection were blocked with 5% bovine serum albumin (BSA). Selective detection of protein was carried out utilizing the following antibodies: anti-DYKDDDDK FLAG (Sigma-Aldrich Cat. #F2555), anti-phospho-Insulin Receptor beta (Santa Cruz Cat. #sc-81500), and anti-beta-Galactosidase directly conjugated to the enzyme HRP (Horseradish peroxidase, Abcam Cat. #ab191357). Goat anti-mouse secondary antibody (Santa Cruz Cat. #sc-2005) was used for identification of phos-IRβ and goat anti-rabbit secondary antibody (Santa Cruz Cat. #sc-2004) was used for identification of 3×FLAG protein. Protein bands were visualized by chemiluminescence *via* Clarity Western ECL substrate (Bio-Rad). Images were analyzed using the Bio-Rad chemi Doc XRS+ image analyzer. Positive control proteins include: recombinant *E. coli* LacZ protein (Abcam) and Amino-terminal FLAG-BAP™ Fusion protein (Sigma-Aldrich).

### Flow cytometry and fluorescence activated cell sorting

Flow cytometry was carried out to identify the presence of surface expressed 3×FLAG tagged mIR protein. Cells were treated with TruStain FcX affinity purified anti-mouse CD16/32 Fc block (BioLegend) and stained with DYKDDDDK (FLAG) Tag specific polyclonal Alexa Fluor^®^ 647 conjugated rabbit antibody, detected in the allophycocyanin (APC) channel (Cell Signaling Technology, cat#3916s). Cell samples were analyzed on a BD FACSCaliber cytometer (BD Biosciences).

In order to make a stable HEK-Cre+ 3×FLAG-mIR expressing cell line, the pPNTlox2-3×FLAG-mIR plasmid was linearized with Afl-II and Spe-I to remove all residual bacterial components (**Fig. S1**). The linearized expression cassette was transfected into HEK-Cre+ expressing cells to allow non-specific homologous recombination to occur. Cells were stained for 3×FLAG tagged mIR protein and sorted to recover eGPF and APC double positive cells. The FACS sorted double positive cells, which will be referred to as the 3×FLAGmIR cell line, were allowed to recover prior to two additional rounds of cell sorting to ensure gene integration and protein expression. Cells were sorted using the BD FACSAria IIu flow cytometer (BD).

### IR stimulation assay

To assess the functionality of the 3×FLAG-mIR and test its ability to initiate proper phos-IRβ, once insulin binds to the IR, an insulin stimulation experiment was performed. Cells were serum starved in 0.01% FBS containing DMEM medium for 16–18 h prior to stimulation. Cells were left unstimulated or stimulated with 100 nM of insulin (Sigma-Aldrich) for 2 min at room temperature. After treatment, the cells were lysed to extract protein for FLAG resin immunoprecipitation and western blot analysis. Insulin stimulated HEK-Cre+ non-immunoprecipitated cell lysate was utilized as the positive control for phos-IRβ detection due to their presence of native IR. Inversely, HEK-Cre+ cell lysates were utilized as a negative control for the detection of DYKDDDDK FLAG phos-IRβ after resin column immunoprecipitation due to their lack of 3×FLAG expression.

### DYKDDDDK FLAG resin immunoprecipitation

Protein extracts from the IR stimulation assay were subjected to immunoprecipitation of DYKDDDDK FLAG-tagged protein *via* an anti-DYKDDDDK G1 affinity resin (GenScript L00432-1) to separate native IR from the 3×FLAG-mIR. Immunoprecipitated proteins were eluted from the resin in PAGE sample buffer *via* boiling.

### IR binding titration

The 3×FLAG-mIR cell line and HEK-Cre+ cells were assessed *via* an IR binding titration assay. Cells were incubated with various sample concentrations of biotinylated-insulin (Dr. Finn, University of Pittsburg, PA) ranging from 1 × 10^–7^ M to 1 × 10^–13^ M, for 45 min at 4°C. Cells were stained with Streptavidin Alexa Fluor^®^ 647 Conjugate (Invitrogen) secondary reagent and analyzed on a BD FACSAriaIIu cytometer.

### Cell immunohistochemistry and imaging

HEK-Cre+ cells and the 3×FLAG-mIR cell line were grown out in 24 well plates on glass slides. The HEK-Cre+ cells were transfected with the pPNTlox2 or the pPNTlox2-3×FLAG-mIR plasmid and stained 48 h post transfection. All samples were incubated with the plasma membrane dye, Cell Navigator™ Cell Plasma Membrane Staining Kit (AAT Bioquest). Following plasma membrane staining, cells were fixed with 4% paraformaldehyde, blocked with 2% BSA, and stained with DYKDDDDK FLAG-tagged specific polyclonal Alexa Fluor^®^ 647 conjugated antibody (Cell Signaling Technology). Cells were mounted to glass microscope slides *via* DAPI Fluoromount-G^®^ (SouthernBiotech) and dried. Image analysis was carried out utilizing a Leica TCS SP5 multiphoton laser scanning confocal microscope (Leica Microsystems) using a 63 × 1.4 NA lens.

## RESULTS

### FVB/NJ-CD3-3×FLAG-mIR/MFM mice analysis

**Figure 1.**
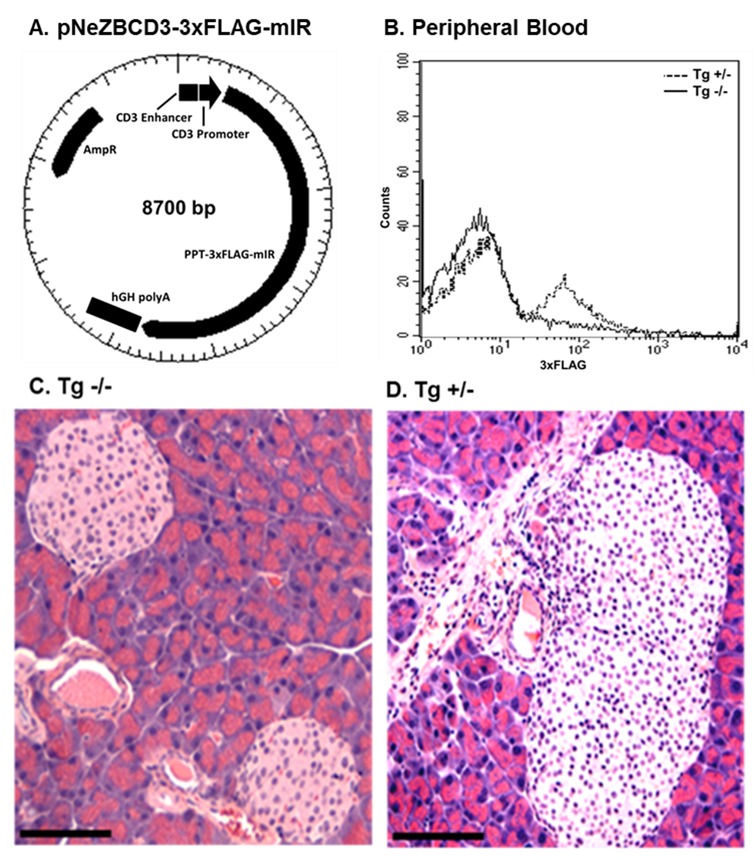
Analysis of FVB/NJ-CD3-3×FLAG-mIR transgenic mice. **A.** Plasmid map of pNeZBCD3-3×FLAG-mIR. **B.** Flow cytometry detection of DYKDDDDK (FLAG) tag specific lymphocytes in peripheral blood of age matched Tg+/- (dotted line) and Tg-/- mice (solid line). **C** and **D.** Pancreatic Islet H&E staining from age matched 6 week old Tg-/- and Tg+/- mice. Images were taken at 20 × magnification, scale bar represents 100 μm.

The pNeZBCD3-3×FLAG-mIR plasmid (**Fig. 1A**) was used to generate FVB/NJ-CD3-3×FLAG-mIR transgenic mice. FVB/NJ-CD3-3×FLAG-mIR mice express a 3×FLAG-mIR transgene insert which encodes a full length mIR containing an N-terminal 3×FLAG tagged α-subunit and full length β-subunit as seen in the native mouse INSR gene. The endogenous signal peptide coding sequence was deleted and replaced with a pre-protrypsin (PPT) signal peptide. The PPT-3×FLAG-mIR transgene was placed downstream of an engineered CD3 enhancer and promoter construct to elicit T-lymphocyte directed transgene over-expression. Tg+/- FVB/NJ-CD3-3×FLAG-mIR mice express detectable 3×FLAG protein on T-lymphocytes found in peripheral blood (**Fig. 1B**, dotted line) and spleen (data not shown). Comparisons of H&E stained pancreatic tissue slides shows lymphocytes in Tg+/- mice compared to age matched 6 week old Tg-/- mice (**Fig. 1C** *vs*. **Fig. 1D**). Heart, liver, lung and kidney were all negative for 3×FLAG expressing T-lymphocytes in either Tg+/- or Tg-/- mice (data not shown).

### pPNTlox2-3×FLAG-mIR plasmid transfection analysis

**Figure 2.**
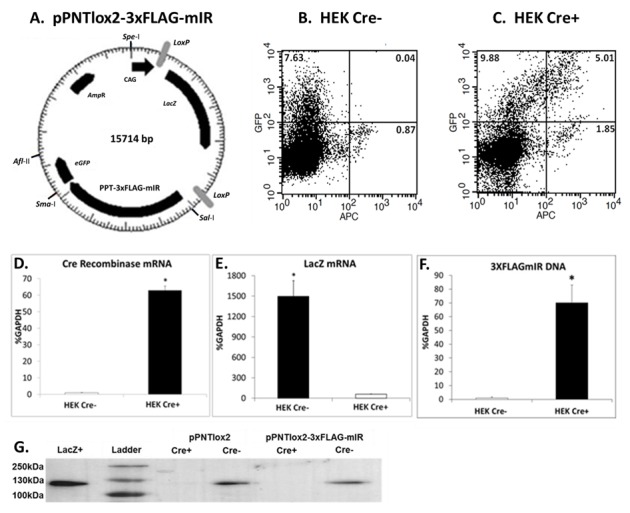
pPNTlox2-3×FLAG-mIR transfection analysis. **A.** Plasmid map of pPNTlox2-3×FLAG-mIR. **B** and **C.** Cytometry detection of FLAG tag specific polyclonal Alexa Fluor^®^ 647 conjugated APC and eGFP *via* transfections with pPNTlox2-3×FLAG-mIR in HEK-Cre- (B) and HEK-Cre+ cells (C). A total of 10000 events were recorded for all samples. Results were confirmed in a total of three separate experiments. **D-F.** qRT-PCR results *via* transfection with pPNTlox2-3×FLAG-mIR in HEK-Cre-(open bar) and HEK-Cre+ cells (solid bars). Samples were performed in triplicate, significance of a *P* value ≤ 0.05 is indicated by an asterisk (*). **G.** Western blot analysis for the detection of LacZ protein in HEK cells transfected with pPNTlox2 or pPNTlox2-3×FLAG-mIR. Equivalent amounts of total protein extract were run in reducing conditions (12 μg) for all experimental samples. Recombinant beta-lactamase protein (LacZ+) was included as a positive control (100 ng). Results were confirmed in a total of three separate experiments.

The PPT-3×FLAG-mIR gene was cloned and sequence verified into the pPNTlox2 parent vector to form the pPNTlox2-3×FLAG-mIR plasmid (**Fig. 2A**). The pPNTlox2-3×FLAG-mIR plasmid contains a CAG promoter driven Cre-Lox based inducible system that expresses the Lac-Z gene as a decoy in the absence of Cre and the mIR gene cassette and a non-covalently linked eGFP reporter in the presence of Cre (**Fig. S1**). Transfection with the pPNTlox2-3×FLAG-mIR plasmid into HEK-Cre- and HEK-Cre+ cells elicits Cre recombination selectively in the Cre+ cells, wherein a distinct population of double positive 3×FLAG-mIR (APC) and eGFP positive cells can be observed by flow cytometry (**Fig. 2B** *vs*. **Fig. 2C**, upper right quadrant).

Additional evidence of selective recombination upon transfection in the presence of Cre recombinase can be seen at both the genetic (**Fig. 2D**-**2F**) and protein (**Fig. 2G**) level. Cre mRNA is only expressed in Cre+ cells (**Fig. 2D**). Ablation of the Lac-Z gene is observed by a decrease of LacZ mRNA expression in HEK-Cre+ cells (**Fig. 2E**). Plasmid recombination in the presence of Cre recombinase was detected in HEK-Cre+ *via* the production of a DNA specific recombination gene product (**Fig. 2F**). Western blot analysis from transfected HEK-Cre+ cells support that a reduction of LacZ mRNA correlates to a reduction in LacZ protein production after transfection with both the pPNTlox2-3×FLAG-mIR and pPNTlox2 plasmids (**Fig. 2G**).

### 3×FLAG-mIR cell line generation and analysis

**Figure 3.**
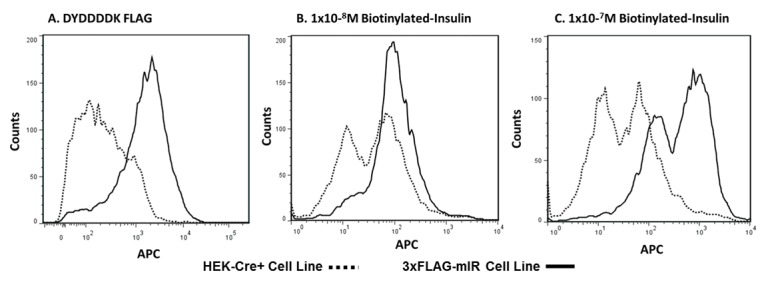
Flow cytometry analysis of the 3×FLAG-mIR cell line. **A.** Flow cytometry detection of FLAG tag specific polyclonal Alexa Fluor^®^ 647 conjugated APC in untransfected HEK-Cre+ cells (dotted line) and the 3×FLAG-mIR cell line (solid line). A total of 10000 events were recorded for all samples. Results were confirmed in a total of three separate experiments. **B** and **C.** Flow cytometry detection of HEK-Cre+ (dotted line) and the 3×FLAG-mIR cell line (solid) incubated with biotinylated-insulin and detected *via* streptavidin-APC. Data represents cells treated with1 × 10^-8^ M (B) 1 × 10^-7^ M (C) biotinylated-insulin. A total of 10000 events were recorded for all samples. Results were confirmed in two titration experiments.

HEK-Cre+ cells were transfected with the linear pPNTlox2-3×FLAG-mIR expression cassette and were sorted three times to isolate the 3×FLAG-mIR cell line which is double positive for the expression of 3×FLAG-mIR and the eGFP reporter. The 3×FLAG-mIR cell line was characterized to determine the expression of 3×FLAGmIR and the ability to bind biotinylated-insulin in comparison to HEK-Cre+ cells (**Fig. 3**). FLAG reactive antibody strongly stained the 3×FLAG-mIR cell line compared to the HEK-Cre+ cells (**Fig. 3A** solid line *vs*. dotted line). The HEK-Cre+ cells and the 3×FLAG-mIR cell line both express native endogenous IR. However, 3×FLAG-mIR cells bind increased amounts of biotinylated-insulin upon insulin titration specifically at concentrations of 1 × 10^-7^ M and 1 × 10^-8^ M compared to HEK-Cre+ cells (**Fig. 3B** and **3C** solid lines *vs*. dotted lines).

**Figure 4.**
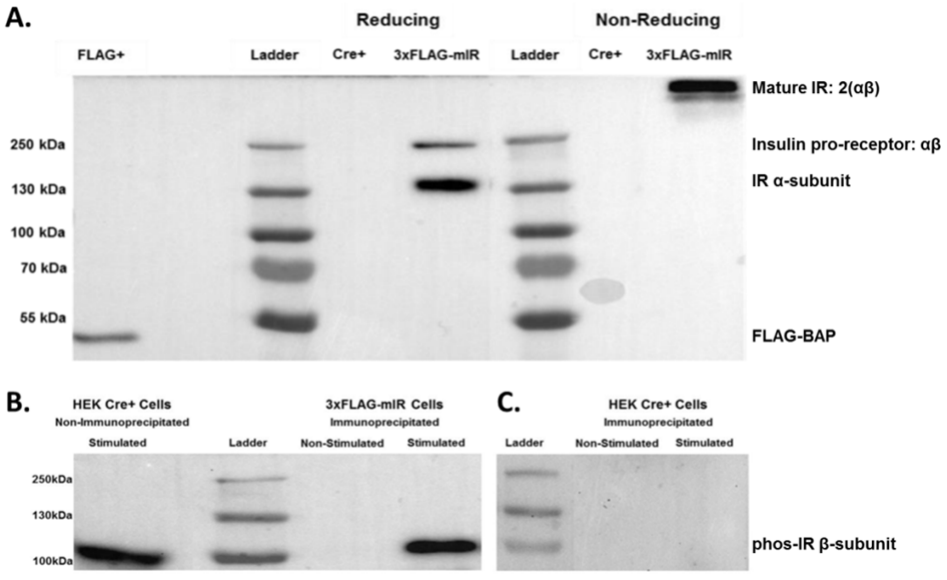
Western blot analysis of the 3×FLAG-mIR cell line. **A.** Analysis of protein from HEK-Cre+ and the 3×FLAG-mIR cell line. Extracted protein was loaded in equivalent amounts (10 μg) for all samples in both reducing and non-reducing conditions and were stained to detect FLAG protein. Reducing conditions identify the 230 kDa Insulin pro-Receptor (αβ) and the 135 kDa mIR α-subunit in 3×FLAG-mIR cells. Non-reducing conditions identify the intact mature 3×FLAG-mIR 2(αβ) heterodimer as a single band above 250 kDa. Recombinant BAP-FLAG positive control protein is detected at 49 kDa. Results were confirmed in two separate experiments, (B) and (C). Analysis of HEK-Cre+ and the 3×FLAG-mIR cell line following 100 nM insulin stimulation. Protein extracts were immunoprecipitated *via* an anti-DYKDDDDK G1 affinity resin. Anti-phos-IRβ specific primary antibody was to detect phosphorylated β-subunit at 95 kDa. **B.** Equivalent amounts of immunoprecitiated proteins were utilized (2.5 μg), while non-immunoprecipitated stimulated HEK-Cre+ cell extract (10 μg) served as the phos-IRβ stimulated positive control. **C.** HEK-Cre+ immunoprecipitated cell extracts produced no detectable band. Results for the generation of specific phos-IRβ stimulated products in 3×FLAG-mIR cells were validated in three separate stimulation and immunoprecipitation experiments.

Western blot analysis specific for 3×FLAG detection reveals recombinant 3×FLAG-mIR protein production in only the 3×FLAG-mIR cell line (**Fig. 4A**). Samples run in reducing conditions identify detectable 3×FLAG tagged α-subunit at 135 kD and the full length 3×FLAG tagged Insulin pro-receptor (αβ) 230 kDa, matching previously reported IR molecular weights [12-14]. Non-reducing conditions detect a single band above the 250 kDa protein marker, which represents the mature fully assembled 3×FLAG-mIR containing two αβ heterodimers matching identified assembled IR molecular weights [12-14].

To selectively assess the ability of the 3×FLAG tagged mIR to bind insulin and undergo proper internal phosphorylation of the IR β-subunit, similar to the native IR [1,15-16] an insulin stimulation assay was performed. Following the insulin stimulation assay cell extracts were subject to immunoprecipitation with a FLAG resin to recover only FLAG expressing proteins prior to detection of phos-IRβ known to be detected at 95kDa [16]. **Figure 3B** and **3C** clearly illustrates that only immunoprecipitated FLAG tagged mIR protein from the 3×FLAG-mIR cell line contains detectable phos-IRβ. HEK-Cre+ cells are able to initiate proper phosphorylated β-subunit upon insulin stimulation prior to resin immunoprecipitation (**Fig. 4B**), but yield no detectable product after immunoprecipitation due to their lack of FLAG expression (**Fig. 4C**).

**Figure 5.**
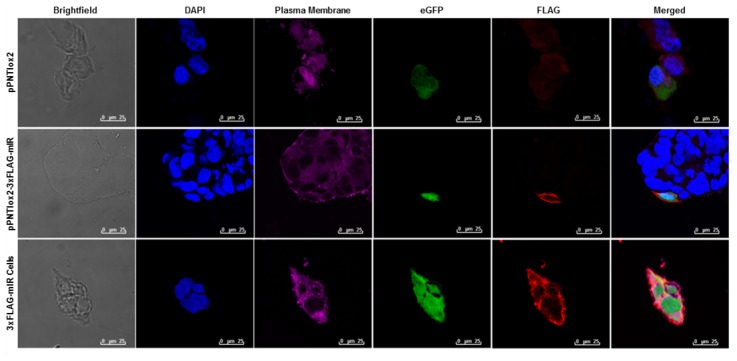
Immunohistochemical analysis of transfected HEK Cre+ and 3×FLAG-mIR cells. The 3×FLAG-mIR cell line and HEK-Cre+ cells transfected with either pPNTlox2 or pPNTlox2-3×FLAG-mIR were stained and imaged *via* laser scanning confocal microscopy. Equivalent staining and detection was carried out for all samples, scale bar is equal to 25 μm. All images display intact cells in the bright field panels, nuclei staining *via* DAPI (blue), and plasma membrane staining *via* Cell Navigator^®^ Plasma Membrane Stain (magenta). The reporter eGFP is displayed in green, while detectable FLAG protein is displayed in red. Control cells transfected with pPNTlox2 can be found in the top panels. Cells transfected with pPNTlox2-3×FLAG-mIR can be found in the middle panels. The 3×FLAG-mIR cell line samples are found in the bottom panels. Staining experiments were repeated twice.

Immunohistochemical staining analysis of transfected HEK-Cre+ and the 3×FLAG-mIR cell line was performed (**Fig. 5**). Equivalent staining intensities and microscope settings were utilized for all images. All images display intact viable cells in the bright field panels, consistent nuclei staining, and detectable plasma membrane staining. Cells transfected with the pPNTlox2 plasmid were used as the negative control. Cells transfected with pPNTlox2-3×FLAG-mIR plasmid detectable eGFP, localized with intense detectable 3×FLAG-tagged protein in a low proportion of cells. The 3×FLAG-mIR cell line displays a dramatic amount of eGFP protein localized with high intensity of detectable 3×FLAG-tagged protein in a high proportion of cells. When overlaid the 3×FLAG-mIR stain and the plasma membrane stain to merge directly on top of one another, providing evidence for 3×FLAG-mIR localization within the plasma membrane.

## DISCUSSION

The IR is the key mediator for cellular insulin stimulation and glucose uptake [17]. The IR spans the plasma membrane of a cell and contains both intracellular and extracellular portions, as well as a transmembrane spanning domain [18-19]. Activation of the IR induces a vast array of physiological and metabolic effects, most notably the uptake of glucose and the conversion of excess glucose into glycogen [2-4,17]. While much is known about the signaling and metabolic role of the IR there is still much to be uncovered about the potential role of the IR in the pathogenesis of T1D.

Previous data from our laboratory shows the importance of IR surface expression levels as a key modulator in lymphocyte directed pathogenesis of T1D [7]. Purified lymphocytes from spleens of diabetic NOD mice were sorted into low and high mIR expressing populations and then adoptively transferred into irradiated recipient mice [7]. Recipient mice that received the mIR high expressing lymphocytes went on to develop insulitis and rapid diabetes while mice that received mIR low expressing transfers did neither [7]. This evidence illustrates a novel hypothesis for high density insulin receptors on the surface of T-lymphocytes as a driving factor for immune trafficking to the pancreas during the progression of T1D.

Based on these findings the FVB/NJ-CD3-3×FLAG-mIR/MFM mouse was developed to assess T-lymphocyte mIR over-expression in a non-autoimmune prone strain of mice. The data collected from the transgenic model illustrates that additional expression of the mIR can cause the presence of T-lymphocytes in the pancreas in a non-antigenic specific chemotactic manner. Although Tg+/- mice expressed detectable 3×FLAG positive lymphocytes in peripheral blood, the over-all CD3 and FLAG specific populations were relatively lower than expected. Tg+/- mice displayed a range of 10.2%–15.8% CD3-FLAG positive lymphocytes in peripheral blood compared to that of roughly 2.5% found in Tg-/- mice (data not shown). The limiting factor for strong induction of mIR over-expression in the FVB/NJ-CD3-3×FLAG-mIR/MFM model is most likely due to the CD3 enhancer promoter construct. The novel findings linking IR over-expression to detectable lymphocytes in the pancreas of a non-autoimmune background sets the stage for a continued effort to determine the physiological implications of IR over-expression in the NOD T1D murine model to determine whether antigen specificity or the chemotactic nature of the IR is driving initial lymphocytes towards the pancreas.

The presented CAG viral promoter driven Cre-Lox system was successfully validated through both transfection and stable gene integration in HEK-Cre+ cells. The gene integration observed *in vitro* provides confidence for use of the CAG transgene in future *in vivo* transgenic animal applications. This system induced cell specific Cre restricted expression of functional 3×FLAG tagged mIR and the associated eGFP reporter. Validation of this system was necessary prior to generation of floxed NOD-3×FLAG-mIR/MFM mice. The floxed NOD-3×FLAG-mIR/MFM mice will be generated for mating with commercially available transgenic NOD mice which selectively expresses Cre in thymocytes during all phases of growth and development (Jax Lab: NOD.Lck-cre). We hypothesize that the progression toward T1D should increase drastically in transgene positive F1 offspring compared to transgene negative controls. Prevention of diabetes can be tested by mating of floxed NOD-3×FLAG-mIR/MFM mice with commercially available NOD.FoxP3-cre mice (The Jackson Laboratory) to test the ability of T-regulatory cells to be delivered therapeutically *via* IR based chemotaxis. In addition to the potential use of these systems in migration and trafficking studies, these IR over-expression systems can be utilized in T1D IR auto-antibody studies to test for the presence of IR specific auto-antibodies and their ability to influence insulin binding and receptor stimulation as previously shown [20].

Understanding what triggers elicit insulitis and lymphocyte migration toward the pancreas during the pathogenesis of T1D holds strong therapeutic value. While antigenic determinants have long been investigated in the development of T1D, other modes of motility, such as chemotaxis, could be strong factors for initial lymphocyte trafficking toward the pancreas. Although the FVB/NJ-CD3-3×FLAG-mIR/MFM mice data strengthen the implications of chemotaxis based T-lymphocyte migration towards the pancreas, there are drawbacks to this system due to the low number of FLAG specific lymphocytes found in the periphery. In order to improve upon these drawbacks, the constitutive viral CAG promoter system will be used in combination with available models that selectively express Cre behind the strong lymphocyte protein tyrosine kinase (lck) promoter to ensure robust generation of high populations of mIR over-expressing T-lymphocytes. Understanding the driving force behind lymphocyte trafficking towards pancreatic islets during the progression of T1D is necessary in the design of novel therapeutic preventatives in the treatment of T1D.

## Supplementary Material

Supplementary information **Table S1**. 3×FLAG-mIR sequencing primers.
**Table S2**. qRT-PCR mRNA and DNA primers.
**Figure S1**. qRT-PCR primer alignment.
Supplementary information of this article can be found online at http://www.jbmethods.org/jbm/rt/suppFiles/209.
